# No Detectable Effect of Caffeine Expectancy Induced by a Placebo Capsule on Time to Exhaustion at the Respiratory Compensation Point in Trained Cyclists

**DOI:** 10.3390/sports14090393

**Published:** 2026-09-04

**Authors:** Fernando Valero, Fernando González-Mohíno, Alejandro Alda-Blanco, Violeta Muñoz de la Cruz, Sergio Rodríguez-Barbero, Miguel Ángel Galán-Rioja, Juan José Salinero

**Affiliations:** 1 Sport Training Lab, Faculty of Sport Sciences, University of Castilla-La Mancha, 45071 Toledo, Spain; fernando.valero1@alu.uclm.es (F.V.); alejandro.alda@uclm.es (A.A.-B.); violeta.munoz@uclm.es (V.M.d.l.C.); sergio.rexposito@uclm.es (S.R.-B.); juanjose.salinero@uclm.es (J.J.S.); 2 Facultad Ciencias de la Salud y Escuela de Doctorado, Universidad Internacional de La Rioja, 26004 Logroño, Spain; miguelangel.galan@unir.net

**Keywords:** placebo, cycling physiology, perceived exertion

## Abstract

This study aimed to analyze the placebo effect associated with caffeine ingestion on cycling performance at the respiratory compensation point (RCP). Nineteen trained male cyclists (age: 28.1 ± 9.4 years; body mass: 71.0 ± 8.4 kg; VO_2_max: 67.1 ± 6.7 mL·kg^−1^·min^−1^) completed two time-to-exhaustion (TTE) tests at the power output associated with RCP under two conditions: caffeine informed/placebo ingested (Expectancy) and non-ingested (Control), separated by 4–7 days. Total work (kJ) was recorded as the main performance variable and heart rate and rating of perceived exertion were measured. The side effects were evaluated through a questionnaire administered the day after the expectancy condition. TTE performance was similar (157.31 ± 63.45 kJ Expectancy vs. 154.83 ± 56.19 kJ Control, *p* = 0.68, trivial *d* = 0.09). Heart rate did not differ between trials (173.05 ± 9.65 bpm vs. 173.32 ± 9.74 bpm, *p* = 0.71 trivial *d* = −0.07), and RPE reached maximal values. Mild side effects—mainly increased activeness and muscular pain (31.3%)—were reported following placebo ingestion. In conclusion, this study did not detect an effect of a caffeine expectancy manipulation on time to exhaustion at the RCP in trained male cyclists. Heart rate was similar between conditions, and mild subjective symptoms were reported following the expectancy condition, although these cannot be causally attributed to expectancy.

## 1. Introduction

The placebo effect refers to a beneficial outcome that follows from a person’s expected and/or learned response to a treatment or situation [1]. It can influence subjective experiences, such as fatigue and illness symptoms, as well as objective measures, such as pain tolerance [2]. These responses can vary and are shaped by environmental, psychological, and neurobiological factors, including verbal cues, expectations, desires, positivity, beliefs, and conditioning [3,4]. At the neurobiological level, placebo-induced expectations may engage reward-related mechanisms involving dopaminergic, opioidergic, and glutamatergic pathways, which may influence motor processes and the perception of effort [5]. Because perceived exertion is a centrally generated construct reflecting the relative rather than absolute demand of an exercise bout [6,7], changes in reward-related expectations could theoretically alter the rating of perceived exertion (RPE) at a given intensity and, particularly in effort-regulated tasks, influence exercise performance. Conversely, the nocebo effect occurs when negative expectations adversely affect performance, for example when individuals believe they have received an inert treatment [8] or have been informed that a treatment may impair performance despite receiving a placebo [9,10].

Interest in the placebo effect as a means of modulating sports performance has grown considerably, with research focusing on nutritional, pharmacological, and mechanical ergogenic interventions. Between 2000 and 2018, 31 studies investigating placebo and nocebo effects on sport performance were identified [11], while a subsequent review identified 20 additional studies [9]. Although these experimental designs are challenging because they frequently involve deception, the available evidence suggests that placebo interventions can exert a small-to-moderate effect on sports performance. Among the substances commonly investigated in placebo research, caffeine stands out as one of the most frequently studied ergogenic aids [8]. This is particularly relevant because caffeine is also one of the most widely used ergogenic aids in sport [12], with evidence supporting its performance-enhancing effects on muscular endurance, strength, anaerobic power, and aerobic performance [13,14]. Moreover, its ergogenic effects appear to be greater during aerobic than anaerobic exercise [15] which may partly explain the high prevalence of caffeine use among athletes participating in endurance disciplines such as rowing, cycling, and triathlon [12].

In cycling, caffeine is considered one of the most consistently supported nutritional strategies for enhancing performance across a wide range of exercise durations, intensities, and distances [16,17,18]. Although its mechanisms are multifactorial, caffeine appears to act primarily as a non-selective antagonist of adenosine receptors in the central nervous system [14,19], increasing neural activation and neurotransmitter release, and potentially influencing motor-unit recruitment and fatigue [19,20,21]. Experimental caffeine studies therefore provide an opportunity to distinguish its pharmacological effects from psychological responses associated with the belief that caffeine has been consumed. Placebo administration can be used to induce such a belief without administering caffeine, thereby enabling expectancy-related responses to be examined in the absence of caffeine pharmacological effects [22,23]. However, the specific effect captured depends on the experimental design and control condition, because placebo administration also incorporates the act of treatment and its associated ritual.

Given the widespread use of caffeine among endurance athletes, expectations derived from previous experience and knowledge of its ergogenic effects could potentially influence performance even in the absence of caffeine’s pharmacological effects [24,25]. Evidence from running supports this possibility. Several studies have reported improved performance when trained runners believed they had consumed caffeine despite receiving a placebo, including 1 km and 4 km time trials [26,27] and a 6 min fixed duration effort [28]. These findings suggest that caffeine-related information and expectancy may influence endurance performance under some experimental conditions, although the magnitude and mechanisms of these responses remain uncertain.

Evidence in cycling, however, is scarce and less consistent [9,25]. Beedie et al. [25] reported modest changes in power output during a 10 km cycling trial when participants believed they had ingested caffeine with the apparent effect varying according to the communicated dose, although the overall group-level effect was not practically meaningful. Similarly, Foad et al. [9] demonstrated an interaction between information and caffeine administration during a 40 km time trial, whereas the main effect of being informed that caffeine had been ingested was only possibly beneficial. Collectively, these findings indicate that caffeine-related expectancy may influence cycling performance under some conditions, but they do not establish a consistent group-level ergogenic effect. Moreover, differences observed between individual trials should not be interpreted as evidence of true “responders” without repeated measurements and a predefined threshold exceeding the protocol’s measurement error.

The distinction between pharmacological and expectancy-related effects is also relevant because caffeine ingestion may produce adverse effects, including nervousness, gastrointestinal discomfort, and sleep disturbance, particularly at moderate-to-high doses [25]. Symptoms commonly associated with caffeine may also be reported following placebo administration when individuals believe that caffeine has been consumed. For example, increased perceived activeness has been reported following a caffeine expectancy manipulation in runners [28]. These findings suggest that expectations may influence subjective responses to an intervention even in the absence of the active substance. However, such subjective responses should not be interpreted as physiological effects of caffeine or assumed to result in corresponding improvements in exercise performance.

Despite the growing interest in caffeine-related expectancy effects, evidence in cyclists has been obtained predominantly from self-paced time-trial protocols, including 10 km and 40 km trials [9,25]. These protocols allow participants to continuously adjust power output and pacing according to perceived effort, motivation, and expectations. In contrast, a time-to-exhaustion (TTE) protocol performed at a fixed power output restrict self-selected pacing adjustments and therefore provides a different experimental context in which to examine exercise tolerance [29]. TTE performance has been associated with cycling time-trial performance in trained cyclists [30] while fixed-intensity TTE protocols have demonstrated acceptable test–retest reliability under specific experimental conditions [31]. The respiratory compensation point (RCP) represents an identifiable physiological threshold during incremental cycling exercise and provides a standardized high-intensity workload for TTE testing. Therefore, performing a TTE test at the power output associated with RCP provides an externally controlled condition that limits the athlete’s ability to modify power output in response to perceived effort or expectations. Whether a caffeine-related expectancy manipulation can influence exercise, tolerance under these constrained conditions has not, to our knowledge, been previously examined.

Accordingly, the aim of this study was to evaluate whether ingesting an inert capsule presented as caffeine influenced TTE at the power output corresponding to RCP in trained cyclists, compared with a no-ingestion control condition. Importantly, because the expectancy condition combined the belief of having ingested caffeine with capsule ingestion and its associated, treatment ritual, the present design evaluates the combined effect of this manipulation relative to no ingestion rather than the belief component in isolating caffeine expectancy itself from the broader placebo response. We hypothesized that the Expectancy condition would not meaningfully extend TTE or total work compared with the Control condition because the externally imposed workload restricted pacing-related behavioral adjustments. As secondary hypotheses, we expected heart rate and RPE to remain similar between conditions and anticipated that participants would report mild subjective symptoms consistent with the belief of having ingested caffeine.

## 2. Materials and Methods

### 2.1. Participants

A power analysis was performed a priori (G*Power software v3.1.9.7; Universität Kiel, Kiel, Germany) based on the effect size observed in the study of Valero, González-Mohíno [28]. This analysis indicated that 19 participants were required to detect a moderate effect size (0.69), with an alpha value (α) of 0.05, and a power (1-β) of 0.8. Therefore, nineteen male trained cyclists were recruited through local cycling clubs (mean ± SD: age 28.1 ± 9.4 years, body mass 71.0 ± 8.4 kg, height 178.2 ± 6.8 cm, body fat 12.0 ± 2.7%, maximal oxygen uptake [VO_2_max] 67.1 ± 6.7 mL·kg^−1^·min^−1^, and maximal aerobic power [MAP] 400.5 ± 42.4 W). The inclusion criteria were the following: (1) participants must engage in regular cycling training with at least four sessions per week during the past year, (2) regularly participate in competitions within their discipline, (3) hold a regional or national cycling license, (4) at least three years of experience in regular cycling training, and (5) have no physical limitations, musculoskeletal injuries, or respiratory or cardiovascular diseases that could impair their performance.

Prior to the study, all participants were informed about the testing protocols and potential risks involved. All subjects participated voluntarily in this study after signing a written informed consent form. The study was conducted in accordance with the principles of the Declaration of Helsinki (October 2024, Helsinki, Finland), and the experimental protocols were approved by the local ethics committee (PI:030_2025, 2025 April). The study was retrospectively registered on the Open Science Framework (OSF; osf.io/hrz8c) 12 May 2026.

### 2.2. Design

A repeated measures, randomized, and counterbalanced experimental design was used to compare the effects of ingesting a placebo reported as caffeine (expectancy condition) and a control condition in which no substance was ingested (control). Participants performed two TTE tests at the power output (PO) corresponding to their RCP under the two experimental conditions separated by an interval of 4 to 7 days.

### 2.3. Procedure

Participants completed three sessions separated by 4–7 days (5.0 ± 0.9 days). In session 1, a graded exercise test was performed to determine the RCP. In sessions 2 and 3, TTE tests were conducted at the RCP under two conditions: expectancy (participants were informed they had ingested caffeine) and control (no ingestion) and administered in a computer-generated randomized, counter-balanced order to avoid any order effect. After participant enrolment, the principal investigator generated the computer-generated random allocation sequence, assigning participants in a 1:1 ratio to one of the two treatment sequences (placebo–control or control–placebo). The same investigator enrolled the participants and assigned them to one of the two treatment sequences. The allocation sequence was not disclosed to participants before assignment. All data collection was performed under similar environmental conditions for each participant, at an altitude of 529 m, with temperatures ranging between 21 and 23 °C and relative humidity between 27% and 34%, with no significant differences between visits (*p* > 0.05). Participants were instructed to avoid caffeine intake and refrain from intense exercise within the 48 h prior to each visit. Twenty-four hours before each experimental test, participants were asked to maintain similar patterns of sleep, diet, and fluid intake. They were also questioned about any intolerances to the capsule components (e.g., gelatin) or to cornflour (used as the placebo substance, described as an excipient mixed with caffeine). In the first session, body mass was measured using a stand-up multi-frequency bioelectrical impedance analyzer (InBody 770; Cerritos, CA, USA). This measurement was conducted to reinforce the credibility of the caffeine expectancy manipulation, as participants were informed that body mass would be used to individualize the caffeine dose (3 mg·kg^−1^). Although an inert capsule was ultimately administered, the assessment of body mass was necessary to strengthen participants’ belief in a personalized caffeine supplementation protocol. In addition, and to enhance the placebo effect, participants were informed about the ergogenic properties of caffeine and its previously demonstrated performance-enhancing effects in cycling and other endurance tests. No familiarization trial for the TTE test at RCP was performed, and habitual caffeine intake was not quantified; participants were only required to abstain from caffeine during the 48 h preceding each visit. No belief or manipulation check was administered to verify that participants accepted the information regarding caffeine ingestion, and no formal debriefing was conducted at the end of the study.

### 2.4. Graded Exercise Test

Participants completed a 5 min warm-up at 50 W, after which the workload was progressively increased by 25 W·min^−1^ until volitional exhaustion [29]. Respiratory variables were measured using a gas analyzer (CPX Ultima Series MedGraphics system, St. Paul, MN, USA), which was calibrated prior to each test (CO_2_ 4.10%; O_2_ 15.92%), while the volume sensor was calibrated with a 3 L syringe. The zirconia O_2_ analyzer had a response time below 0.80 ms with an accuracy of ±0.03%, while the CO_2_ analyzer showed a response time under 130 ms and an accuracy of ±0.10%.

To confirm the achievement of VO_2max_, at least two of the following criteria were required [32]: (a) stabilization of VO_2_ values (i.e., an increase of less than 1.5 mL·kg^−1^·min^−1^ across two or more consecutive stages), (b) a respiratory exchange ratio (RER) ≥ 1.10, or (c) reaching a maximal heart rate (HRmax) greater than 95% of the age-predicted value (207 − 0.7 × age). Once verified, MAP was defined as the first workload at which VO_2max_ was attained where “*Wf*” is the last fully completed workload (W) and “*t*” is the time in seconds that the last uncompleted workload was maintained [33]. The gas exchange threshold (GET) was identified by a rise in both the ventilatory equivalent for oxygen (VE/VO_2_) and the end-tidal partial pressure of oxygen (PetO_2_), without a simultaneous increase in the ventilatory equivalent for carbon dioxide (VE/VCO_2_). The RCP was determined by a concurrent rise in VE/VO_2_ and VE/VCO_2_ along with a decrease in the end-tidal partial pressure of carbon dioxide (PetCO_2_) [29,34,35]. For both GET and RCP determination, the values of gas-exchange parameters were averaged every 1 min and plotted against workload. Two researchers (F.V. and F.GM.) individually checked the GET and RCP, and any discrepancies were resolved by consensus.

### 2.5. Time to Exhaustion Test at RCP

Participants performed two TTE tests at the PO corresponding to their RCP under two experimental conditions: expectancy condition, in which participants ingested an inert placebo presented as caffeine, and a control condition with no substance ingestion. On the experimental test days, each participant arrived at the laboratory with their own bicycle and regular cycling equipment. Before the expectancy condition test, participants were given a gelatin capsule (size 00, Guinama, Valencia, Spain) containing a placebo substance (100 mg of cornflour) and were told it contained 3 mg·kg^−1^ of caffeine, which they were instructed to ingest with 250 mL of water 60 min before the time-to-exhaustion test at RCP. In the control condition, the participants were not given anything.

The protocol began with a 10 min warm-up, consisting of 5 min at 80% and subsequently 5 min at 90% of the PO associated with the GET [36]. The test finished when the pedaling cadence dropped below 60 revolutions per minute (rpm) [37]. To assess maximal effort, the rating of perceived exertion (RPE, 0–10 scale) was collected immediately after each trial. Performance was quantified as PO multiplied by time to exhaustion, normalized to kilojoules PO*time to exhaustion/1000 (in kJ) [36,37].

All tests were carried out in hyperbolic mode (i.e., the work rate was imposed on the participants with a constant load independently of the pedaling cadence) using the participants’ own bicycles mounted on a Cycleops Hammer ergometer [38]. Each cyclist selected their preferred pedaling cadence and was blinded to performance-related variables such as elapsed time and heart rate. In both experimental conditions, the same evaluator supervised the exercise sessions and provided standardized verbal encouragement to promote consistent effort across trials. The evaluators were not blinded to condition assignment, as they were responsible for administering the capsule and the corresponding information for the expectancy manipulation. To minimize potential performance bias, they followed a standardized encouragement protocol, using the same predetermined verbal cues and timing in both conditions. Test termination was based on predefined objective criteria (inability to maintain the prescribed cadence) rather than evaluator judgment, thereby promoting consistency across trials. In addition, heart rate was continuously recorded throughout the TTE test using an H10 chest strap (Polar Electro Oy, Kempele, Finland) connected to a Garmin Forerunner 55 watch (Garmin, Olathe, KS, USA), and mean heart rate during the TTE test was used for subsequent analysis.

### 2.6. Side Effects Questionnaire

Finally, the morning after the expectancy condition session, the participants completed an online questionnaire on possible side effects (Google Forms), where they indicated on a dichotomous scale (yes/no) the presence or absence of each possible side effect, such as nervousness, digestive problems, or difficulty sleeping, and rated the intensity of these effects on a scale from 1 (minimal/absence of the symptom) to 10 (maximum intensity), regardless of their yes/no response to the presence of that symptom. This questionnaire was previously used to assess the side effects of caffeine intake in sports [39]. Three participants failed to complete the online questionnaire; therefore, 16 participants were included in this analysis. The questionnaire was administered only following the expectancy condition.

### 2.7. Statistical Analysis

The results from each test were analyzed using the statistical software SPSS v29.0 (IBM Corp., Armonk, NY, USA). Data are presented as means ± standard deviations, while presence of perceived side effects was reported as frequencies. Standard deviations are reported as descriptive indicators of between-participant variability in absolute work capacity. First, the normality of each variable was assessed using the Shapiro–Wilk test. A 2-way repeated-measures ANOVA was used to analyze the potential interaction between condition (within-subject factor) and sequence order (between-subject factor; expectancy-control vs. control-expectancy). For all statistical tests, a significance level of *p* < 0.05 was set to determine statistically significant differences. Additionally, effect sizes for the pairwise comparisons (control vs. expectancy) were calculated using Cohen’s *d*, interpreted as follows: <0.20 trivial, ≥0.20–0.59 small, ≥0.60–1.19 moderate, ≥1.20–1.99 large, and ≥2.00 very large, in line with Hopkins’ recommendations [40]. No formal equivalence test was performed; accordingly, the non-significant condition effect reported below is interpreted as a failure to reject the null hypothesis rather than statistical confirmation of equivalence between conditions [41].

## 3. Results

**Figure 1:**
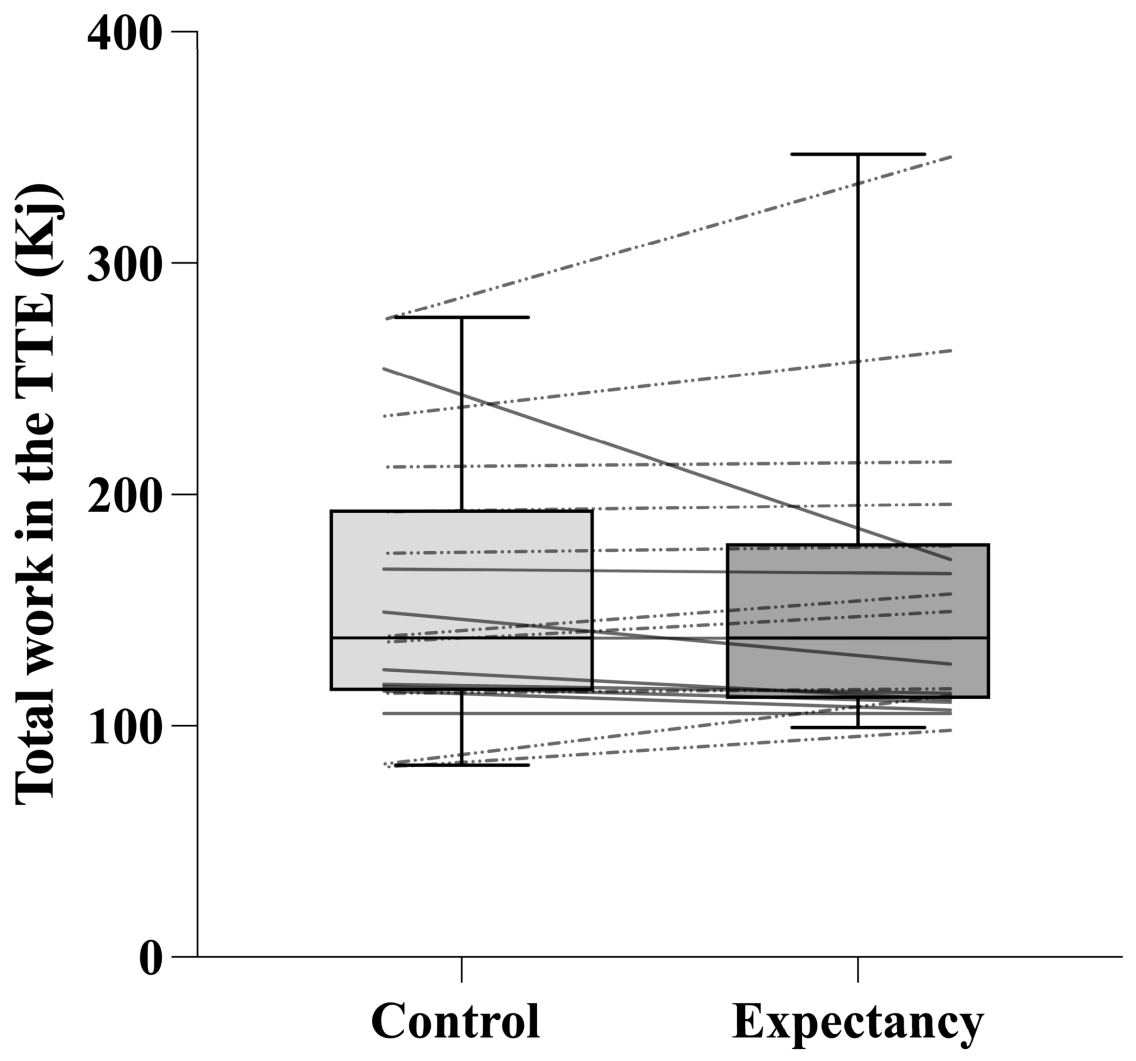
Total work in Time-to-Exhaustion Performance Under Expectancy and control condition. Note: Total work (kJ) achieved in the time-to-exhaustion test (TTE) under the expectancy and control conditions. Each line represents an individual participant’s performance across both experimental conditions. Solid lines correspond to participants whose total work was higher in the control condition, whereas dashed lines indicate those whose total work was higher in the expectancy condition.

No significant differences were observed between the Expectancy and Control conditions for total work performed during the TTE tests (157.31 ± 63.45 vs. 154.83 ± 56.19 kJ, respectively; mean difference = 2.48 kJ, corresponding to a 1.60% higher value in the Expectancy condition, 95% CI [−11.17, 16.80]; F (1,17) = 0.18, *p* = 0.676, η^2^p = 0.011), with a trivial effect size (d = 0.09; Figure 1). The condition × sequence interaction was not significant (F (1,17) = 0.93, *p* = 0.348, η^2^p = 0.052), indicating that the order in which participants completed the Expectancy and Control conditions did not influence total work performed during the TTE tests. As shown in Figure 1, some participants had higher total work in the Expectancy condition relative to Control; however, given the absence of a predefined meaningful-change threshold exceeding the protocol’s measurement error, these individual differences should not be interpreted as improvement. Similarly, time to exhaustion at the RCP (335.53 ± 25.43 W) did not differ significantly between the Expectancy and Control conditions (466.21 ± 172.34 vs. 459.05 ± 153.29 s, respectively; mean difference = 7.16 s, corresponding to a 1.55% higher value in the Expectancy condition, 95% CI [−31.90, 48.17]; F (1,17) = 0.18, *p* = 0.674; η^2^p = 0.011), also showing a trivial effect size (d = 0.09). The condition × sequence interaction was likewise not significant for time to exhaustion (F (1,17) = 0.95, *p* = 0.342, η^2^p = 0.053), indicating that the order in which participants completed the Expectancy and Control conditions did not influence this outcome either.

In the same way, mean heart rate during the TTE was nearly identical between conditions (173.05 ± 9.65 vs. 173.32 ± 9.74 bpm; mean difference = −0.32 bpm, 95% CI [−2.14, 1.50]; F (1,17) = 0.14, *p* = 0.71, η^2^p = 0.008), with a trivial effect size (*d* = −0.07). RPE reached the ceiling value (10 on a 0–10 scale) in both conditions.

The side-effect questionnaire was administered once, following the expectancy condition, and was completed by 16 participants; therefore, these findings reflect symptoms reported after this condition rather than changes attributable to the expectation itself. The most frequently reported symptoms were activeness (31.3%; 3.13 ± 2.13) and muscular pain (31.3%; 2.69 ± 2.15), followed by increased urine production (18.8%; 2.19 ± 1.28). Only one out of 16 participants reported nervousness (6.3%; 1.94 ± 1.39), insomnia (6.3%; 1.81 ± 1.56), and headache (6.3%; 1.19 ± 0.54). No cases of irritability or gastrointestinal discomfort were reported (Table 1).

## 4. Discussion

The aim of this study was to evaluate the effect of caffeine expectancy on time to exhaustion at the RCP. The main findings were: (a) no significant difference in TTE was detected between the Expectancy and Control conditions; (b) heart rate responses during the TTE test were comparable between conditions; and (c) mild subjective symptoms were reported by some participants following the Expectancy session.

Our study did not detect a significant difference in TTE at the RCP between the Expectancy and Control conditions; however, this null finding should be interpreted cautiously given the sample characteristics and size. Comparable performance was observed in both trials, though this may reflect the specific conditions of this investigation rather than a generalizable finding. This finding contrasts with previous studies reporting expectancy-related performance effects under certain experimental conditions, such as those by Beedie, Stuart [25] and Foad, Beedie [9]. In these studies, participants were informed that they had ingested moderate-to-high doses of caffeine (4.5 and 9 mg·kg^−1^ in Beedie, Stuart [25]; 5 mg·kg^−1^ in Foad, Beedie [9]). In the present study, and a measurable placebo effect was observed under certain conditions. In our case, participants were informed that they had ingested a moderate dose (3 mg·kg^−1^). Although lower than the doses communicated in previous cycling studies, this dose falls within the range typically considered ergogenic when caffeine is consumed [14]. Participants were also explicitly informed of its performance-enhancing effects to reinforce the expectancy manipulation.

From a mechanistic perspective, placebo-induced expectations have been proposed to engage reward-related pathways, including dopaminergic and opioidergic mechanisms, that may influence the perceived cost of effort [5] and the relationship between perceived exertion and anticipated exercise duration [6,7]. In self-paced tasks, such changes could potentially influence performance through continuous adjustments in power output or pacing according to perceived exertion. In an externally imposed TTE protocol such as that used in the present study, however, power output is fixed, thereby restricting pacing-related adjustments. Under these conditions, an expectancy-related improvement in performance would therefore require an extension of exercise tolerance at the prescribed workload, potentially through altered perceptual or motivational processes. Although this provides a plausible explanation for why expectancy effects may differ between self-paced and externally controlled exercise, the present study did not directly compare these exercise paradigms, and this interpretation should therefore be considered speculative.

Although the externally controlled nature of the TTE protocol may partially explain the absence of a detectable expectancy-related effect, this interpretation should be considered speculative in light of our methodological constraints. Other methodological factors should also be considered, including the absence of a manipulation check and TTE familiarization, the lower communicated caffeine dose compared with previous cycling studies [9,25] and the lack of information on habitual caffeine intake. These factors may have influenced the expectancy response and/or TTE performance and therefore limit our ability to definitively attribute the present findings to any single mechanism.

In other exercise modes, such as running, several studies have reported performance improvements when participants believed they had ingested caffeine, using short-distance time trials (1 and 4 km) and fixed-duration maximal efforts [26,27,28]. These findings are broadly consistent with cycling studies by Beedie, Stuart [25] and Foad, Beedie [9], in which expectancy-related performance effects were observed under certain experimental conditions. Therefore, previous evidence from running and cycling suggests that caffeine-related expectancy may influence endurance performance under some conditions. However, the present study did not detect any significant improvement in TTE at the RCP. Differences in task characteristics may partly explain these contrasting findings. Whereas previous studies primarily employed self-paced time trial or fixed-duration performance tests that allowed participants to regulate their effort, the present study used a TTE protocol at a fixed power output. The externally imposed workload restricts opportunities for pacing adjustments and behavioural self-regulation, which may influence the manifestation of expectancy-related performance effects.

Mean heart rate did not differ significantly between the Expectancy and Control conditions, with a trivial effect size. However, this finding should be interpreted cautiously, as heart rate represents only one marker of physiological strain and does not fully capture the multidimensional nature of internal load. The similar mean heart-rate responses between conditions are consistent with previous cycling [9,25] and running studies [26,27,28], reporting no significant differences between expectancy and control conditions. Given that no changes were observed in performance outcomes or total exercise duration between conditions, it is reasonable that internal load, as reflected by heart rate, remained stable. In this context, expectancy effects, in the absence of alterations in external load, appear insufficient to elicit detectable changes in internal load during maximal or near-maximal exercise. In addition, regarding the RPE, all participants reached the maximum score of 10 on the 0–10 scale, as the protocol was a TTE test. However, maximal RPE at task termination should not be interpreted as independent evidence that participants achieved an equivalent maximal physiological effort in both conditions. Participants terminated the effort either when they could no longer continue or when cadence dropped below 60 RPM, under the supervision of the researcher. Although these predefined termination criteria were applied consistently across all experimental conditions, they cannot be considered evidence that participants reached equivalent maximal physiological effort across conditions. Furthermore, terminal RPE values of 10 should be interpreted cautiously, as the ceiling of the scale limits its ability to detect potential differences in perceived exertion between conditions.

Regarding subjective symptoms, some participants reported increased activeness and muscular pain (31.3% each), following the Expectancy session, followed by increased urine production (18.8%). However, because the side-effects questionnaire was administered only after the Expectancy condition, these symptoms cannot be causally attributed to expectancy, as no comparable assessment was performed after the Control condition. Moreover, some symptoms, particularly muscular pain, may reflect the physical demands of the maximal cycling effort rather than the belief of having ingested caffeine. Valero, González-Mohíno [28] reported broadly similar subjective symptoms under an expectancy condition using a comparable questionnaire, although the present findings indicate that mild subjective symptoms were reported following the Expectancy condition but do not establish whether they resulted from expectancy, exercise itself, or both.

The study presents several limitations that should be acknowledged. First, the sample consisted exclusively of trained men, which limits the generalizability of the findings to other populations, particularly given potential sex-related differences in placebo responses [42]. Second, precise control of participants’ pre-session dietary intake was not feasible. Although participants were instructed to replicate their habitual eating patterns before each trial, minor variations in nutritional status may have influenced performance outcomes. Other methodological limitations also constrain interpretation of the null finding. No manipulation check was administered, so we cannot confirm that the expectancy manipulation was effective, and an unsuccessful manipulation remains a plausible explanation for the absence of an effect. Habitual caffeine intake was not assessed, precluding evaluation of habituation or withdrawal as moderators. No familiarization session with the TTE protocol was conducted. In addition, evaluators supervising the sessions were not blinded to condition assignment, which represents a potential source of performance bias despite the use of standardized encouragement and objective termination criteria. Side effects were assessed only following the expectancy condition, precluding a within-subject comparison of symptom occurrence and intensity between conditions. Finally, no formal debriefing was conducted at the end of the study. Taken together, these limitations underscore that this design cannot separate the specific contribution of belief from that of capsule ingestion and the associated treatment ritual, and this distinction should be borne in mind when interpreting the present findings.

In terms of practical applications and given that this is one of the first studies to examine the effect of caffeine expectancy on a fixed-power time-to-exhaustion protocol, these findings should be considered preliminary. Nonetheless, they call for caution when recommending expectancy-based strategies alone as an alternative to actual caffeine supplementation in this type of protocol. The mild subjective symptoms observed following the expectancy condition suggest that such manipulations may still influence athletes’ perceptual experience, warranting further exploration of their role, particularly in self-paced exercise formats, before firmer practical recommendations can be established.

## 5. Conclusions

In conclusion, this study did not detect a significant effect of caffeine expectancy on time to exhaustion at the respiratory compensation point in trained male cyclists. Heart rate was similar between conditions, and mild subjective symptoms were reported following the expectancy condition, although these cannot be causally attributed to expectancy. Given the sample size and the absence of a manipulation check, these findings should be interpreted as a failure to detect an effect rather than definitive evidence that caffeine expectancy has no effect. Although the externally imposed nature of the TTE protocol may have contributed to these findings, this possibility remains speculative and requires direct comparison with self-paced exercise protocols.

## Figures and Tables

**Table 1:** Side effects experienced by participants in expectancy condition.

	Expectancy	%
Nervousness	1.94 ± 1.39	6.3
Activeness	3.13 ± 2.13	31.3
Irritability	1.44 ± 0.89	0.0
Muscular pain	2.69 ± 2.15	31.3
Headache	1.19 ± 0.54	6.3
Gastrointestinal discomfort	1.00 ± 0.00	0.0
Increase urine production	2.19 ± 1.28	18.8
Insomnia	1.81 ± 1.56	6.3

Note. Prevalence is reported as n (%) based on the 16 participants who completed the questionnaire. Mean intensity was calculated from all 16 respondents, using a scale from 1 (minimal/absence of the symptom) to 10 (maximum intensity).

## Data Availability

The datasets used and/or analyzed during the current study are available from the corresponding author upon reasonable request.
